# Countercurrents: DCIS or Cancer? Why All the Confusion?

**DOI:** 10.3390/curroncol29070392

**Published:** 2022-07-13

**Authors:** Steven A. Narod, Victoria Sopik

**Affiliations:** 1 Women’s College Research Institute, Women’s College Hospital, Toronto, ON M5S 1B2, Canada; victoria.sopik@wchospital.ca; 2 Institute of Medical Science, University of Toronto, Toronto, ON M5S 1A8, Canada; 3 Dalla Lana School of Public Health, University of Toronto, Toronto, ON M5T 3M7, Canada

**Keywords:** DCIS, breast cancer, malignancy

## Abstract

At present, women with ductal carcinoma in situ are counseled that they have a pre-malignant condition which carries the possibility of progression to a fully malignant breast cancer. However, in most cases, the treatment of DCIS resembles that of a small invasive breast cancer and this is a source of confusion to many. In order to properly evaluate the benefit of radiotherapy, mastectomy and contralateral mastectomy, it is necessary to consider the risks of ipsilateral invasive cancer and of contralateral breast cancer in women with DCIS and with small invasive breast cancer. Several registry-based studies indicate that the risks of ipsilateral and contralateral cancer are similar in the two conditions and therefore a similar approach to treatment is rational.

In a recent large survey of 2432 women who were diagnosed with DCIS in the United States, many of the respondents reported confusion—specifically, about their chances of developing an invasive breast cancer, or metastatic cancer, and the options that were available to them to avoid these unwanted outcomes [1]. The women were told that DCIS is not cancer, but it holds the possibility of becoming a cancer. If DCIS is not cancer, then why treat it as such? It is hard for patients (and many doctors) to reconcile the notion that DCIS is not cancer—and lacks the ability to spread—with the fact that it is treated in much the same way as a small invasive cancer. Surgical options include lumpectomy alone, lumpectomy with radiotherapy, and unilateral or bilateral mastectomy [2]. Chemotherapy is off the table, but radiotherapy is a standard of care [2]. Less is known about the benefit of hormonal therapy (e.g., tamoxifen) for the treatment of DCIS. Hoping to minimize the confusion, studies are underway whereby DCIS is treated expectantly, and surgical intervention is offered in the case of tumor growth or progression to invasive disease [3].

We think that much of the confusion is unnecessary and is generated by the current paradigm that separates DCIS and invasive cancer into two distinct conditions. No doubt this position is taken by doctors to reassure patients, relay success (“we got it early”) and reduce anxiety. The position is based on the low mortality rate of DCIS and the belief that stromal invasion is a prerequisite to metastases. After reviewing the clinical literature, we conclude that DCIS is better described as a small breast cancer [4]. Small, but a breast cancer nonetheless.

We base this interpretation on the observation that the mortality of DCIS is 3% over 20 years and that prevention of local invasive recurrence post-DCIS does not reduce breast cancer mortality [5]. If invasive recurrence were the culprit, then surely preventing it should prevent death. There are several papers showing that preventing invasive breast cancer post-DCIS does not prevent death [5,6,7,8,9,10,11]. To our knowledge, there is no paper to the contrary.

Consider the goals of treatment of DCIS. The primary goal is to prevent invasive cancer. Secondary goals are to prevent contralateral cancer, distant recurrence and death from breast cancer. We also want to minimize morbidity and maintain quality of life. Are the goals any different than for women with a small invasive breast cancer? For example, for those women who have a cancer that is less than one centimeter and is node-negative?

Consider the following four points:

1. The risk of an invasive in-breast recurrence following DCIS is the same as the risk of an invasive in-breast recurrence following invasive breast cancer.

In the Banting database, the 15-year risk of invasive local recurrence following DCIS was 15.6%, following stage I breast cancer this was 15.3%, and following stage II breast cancer it was 15.9% [12];

2. The benefit of radiotherapy on reducing ipsilateral invasive breast cancer is the same for patients with DCIS and stage I/II breast cancer.

In the Banting database, the 15-year risk of ipsilateral invasive recurrence was 14% for DCIS patients who received radiotherapy and 29% for DCIS patients who did not receive radiotherapy—a difference of 15% [12]. In the Banting database, the 15-year risk of ipsilateral invasive recurrence was 14% for Stage I/II patients who received radiotherapy and 27% for Stage I/II patients who did not receive radiotherapy—a difference of 13% [12]. Similarly, the benefit of unilateral mastectomy versus lumpectomy (with or without radiotherapy) on preventing ipsilateral invasive recurrence is the same for DCIS patients as it is for early-stage breast cancer patients [5,13];

3. The risk of contralateral invasive breast cancer is the same following DCIS and following invasive breast cancer.

In our SEER-based analysis of 812,851 women with breast cancer, the 25-year actuarial risk of contralateral invasive breast cancer was 10.1% for patients with DCIS and 9.9% for patients with invasive breast cancer [14]. Based on this finding, the rationale for performing a contralateral mastectomy at the time of diagnosis is the same for patients with DCIS as for patients with invasive cancer;

4. Preventing invasive in-breast recurrence after DCIS does not prevent death from breast cancer.

In our SEER based study of DCIS patients, the risk of invasive in-breast recurrence cancer was much higher for women who were treated with lumpectomy than those who were treated with mastectomy (1.3% versus 3.3% at ten years), but the risk of dying of breast cancer was similar [5]. Similarly, the risk of invasive in-breast recurrence cancer was much higher for women who were treated with lumpectomy than those who were treated with lumpectomy and radiation (2.5% versus 4.9%), but the risk of dying of breast cancer was similar. This is similar to what is seen with early-stage invasive breast cancers. In the NSABP randomized trial of women with invasive breast cancer, the risk of invasive in-breast recurrence was much higher for women who had a lumpectomy alone (39%), compared to those who had a lumpectomy plus radiotherapy (14%) or mastectomy (nearly zero), but the risk of dying of breast cancer in the three groups was similar (46%, 46% and 47%, respectively) [15];

5. Chemotherapy is given based on the risk of distant recurrence.

We give women chemotherapy to prevent distant recurrence, not local recurrence. The choice of giving chemotherapy to a woman is dependent on her calculated risk of distant recurrence or death. All agree that the risk of death following DCIS is too low (3%) to warrant chemotherapy—but this does not mean it is not cancer. The risk of dying of cancer for post-menopausal women with small low-grade node-negative breast cancer may also be too low to warrant chemotherapy—but this does not mean that these are not cancers. If we had a chemotherapy regime that was completely non-toxic and without side effects, we would give it to DCIS patients. The decision to give chemotherapy is based on factors other than the presence of cancer cells in the breast stroma. This is one consideration; others include the patient’s age, nodal status, ER status, MammaPrint scores, co-morbidity, etc. It is interesting that among young Black women, the risk of dying of DCIS approaches 10%, and it is possible that some of these women might benefit from chemotherapy [5]. There are other invasive “ultralow risk” breast cancers where the risk of mortality is much lower than this [16].

DCIS, like invasive breast cancer, is heterogeneous; the risks of the various outcomes vary according to host factors, clinical stage and biological subtypes. In order to decide upon treatment for any given patient, the risks of the local and distant recurrence must be considered against the side-effect profile and the morbidity of the treatment under consideration. In the context of DCIS, the protective effect of adjuvant radiotherapy on ipsilateral invasive recurrence is less evident in very young women (diagnosed before age 40) compared to older women [17], whereas for invasive cancer, radiotherapy appears to benefit women of all ages similarly [13]. Despite the heterogeneity in prognosis and treatment response between patients, DCIS exists on a gradient with invasive cancer, sharing fundamental qualities relating to metastatic potential and opportunities for prevention.

The risk of invasive ipsilateral invasive in-breast recurrence is the same for both groups of patients [12], but we do not consider mastectomy as overtreatment of invasive breast cancer. We accept lumpectomy as standard of care for invasive breast cancer, even though the risk of invasive ipsilateral recurrence is much higher after lumpectomy than after mastectomy [5,6,7,8,9,10]. Women seem to worry less about local invasive recurrence after invasive cancer than after DCIS, even though the risks are the same. Perhaps this is an example of the anchoring heuristic. Some consider contralateral mastectomy as an option for women with invasive cancer, but as overtreatment for women with DCIS, even though the risk of contralateral breast cancer is almost exactly the same [14].

The use of radiotherapy has the same impact on reducing the risk of ipsilateral invasive for patients with DCIS and stage I/II breast cancer [13,18] but we are more likely to describe it as overtreatment for DCIS patients.

There is an important point where the statistics on DCIS and invasive cancer diverge. Consider the ten-year risk of distant recurrence or death within 10 years of diagnosis, by patient group:

❖ Following pure DCIS 1.4% [18];
❖ Following DCIS with micro-invasion 2.8% [19];
❖ Following a local invasive recurrence after DCIS 10% [12];
❖ Following a local invasive recurrence after a stage I invasive cancer 25% [12];
❖ Following a local invasive recurrence after a stage II invasive cancer 47% [12].

The key point here is that the mortality rate of a woman who experiences a local invasive recurrence is highly dependent on her initial diagnosis and treatment. Death is much less likely if a DCIS patient experiences a local recurrence than if a stage II breast cancer patient experiences a local recurrence. However, lumpectomy is considered as standard of care for both. Now comes the paradox. We consider a local recurrence after an invasive cancer to be a marker of tumor aggressivity, but not a source of metastases (this was the conclusion of the Fisher NSABP studies and provides the basis for breast-conserving surgery). The invasive recurrence was not the cause of death. Conversely, conventional wisdom says that an invasive recurrence following DCIS is a source of metastases and is the cause of death for the unfortunate few patients who die. So, if we accept conventional wisdom, then there is greater justification to perform a mastectomy for a DCIS patient than for a patient with invasive cancer. No wonder the patients are confused. Aren’t you? Consider if you had two skin lesions and one was more likely to turn malignant, which one would you remove?

In the paper by Rosenberg et al., the patients expressed a great deal of confusion about the risks for local and distant recurrence [1]. Much of the confusion, I think, is generated by the notable lack of correlation between the risk of local recurrence and the risk of distant recurrence across different patient groups. The paradox can be resolved if we consider both types of cancer to be different points on the spectrum. Breast cancer is heterogeneous; DCIS is one end of the spectrum. Accepting this fact will make the rationale behind treatment decisions easier to explain. We should counsel women that DCIS is a small breast cancer with a high risk of recurrence, but thankfully, with a low risk of dying. For women with a small invasive cancer, the specific risks of recurrence and death depend on the specific features and context of the disease, and the treatment selection varies accordingly. The paradigm is relevant for DCIS patients; the risk of dying after DCIS is low on average, varies within and across subgroups, and is not mitigated by local control.

We hope that this analysis reduces confusion; however, it is unlikely to decrease anxiety. This is a separate problem. In a recent study from Princess Margaret Hospital, we showed that the level of anxiety and distress experienced by a woman with a new diagnosis of breast cancer was similar across all stages—despite large differences in prognosis [20].

Based on the risks and patterns of the relevant outcomes described here, we propose that we should counsel women with DCIS as if they have a small breast cancer. We already treat them as such. Ultimately, to optimize the treatment of DCIS and early invasive breast cancer we must differentiate between the cancer processes in the breast and in the systemic niche. Preventing local recurrences will not have the hoped-for downstream effect of preventing deaths; therefore, the de-escalation of local treatment is rational. To prevent death, we must identify and target the subgroup of patients at the highest risk with effective systemic treatments.

The other option is to change the definition of cancer. The NCI defines cancer as “a disease in which abnormal cells divide without control and can invade nearby tissues. Cancer cells can also spread to other parts of the body through the blood and lymph systems.” If we look closely at DCIS (and early-stage lesions), we realize that not all have the capacity to invade and metastasize [4]—some do and some don′t. Time for a new definition of cancer?
